# “Where does the high road lead?” Potential implications of cannabis legalization for pediatric injuries in Canada

**DOI:** 10.17269/s41997-018-0137-3

**Published:** 2018-09-27

**Authors:** Mojgan Karbakhsh, Jennifer Smith, Ian Pike

**Affiliations:** 1 BC Injury Research and Prevention Unit, BC Children’s Hospital Research, F508 4480 Oak St, Vancouver, British Columbia V6H 3V4 Canada; 2 Department of Community Medicine, Tehran University of Medical Sciences, Tehran, Iran; 3 The Community Against Preventable Injuries, Vancouver, British Columbia Canada; 4 Department of Pediatrics, University of British Columbia, Vancouver, British Columbia Canada

**Keywords:** Cannabis, Marijuana, Legalization, Pediatric, Injuries, Child, Adolescent, Cannabis, Marijuana, Légalisation, Pédiatrie, Blessures, Enfant, Adolescent

## Abstract

The purpose of this commentary is to discuss how legalization of non-medical marijuana (LNMM) in Canada can potentially influence child and adolescent unintentional injuries based on evidence from states (American) and jurisdictions that have already legalized cannabis for recreational purposes. Although the evidence is still not conclusive, LNMM can bring about higher exposure, lower perceived harms, and higher prevalence of cannabis use by minors through role modeling and normalization of behaviour within the household and the community, and higher rates of driving under the influence of cannabis, which can contribute to a higher burden of road traffic injuries. Experience of American states with LNMM shows higher rates of emergency visits for pediatric poisoning due to unintentional ingestion of cannabis-containing foods and severe burns due to explosions during the course of home-based cannabis extraction. While the justification for legalization has created a strict legal framework for improved control of cannabis in Canada, the implications for health and safety of children and adolescents necessitate further study, communication with policy-makers and public health practitioners, and evidence-based education of parents, caregivers, and youth.

## Résumé

Notre commentaire porte sur l’influence possible de la légalisation de la marijuana à des fins non médicales (LMFNM) au Canada sur les blessures non intentionnelles subies par les enfants et les adolescents, d’après les données probantes d’États (américains) et d’entités administratives ayant déjà légalisé le cannabis à des fins récréatives. Ces données ne sont pas encore concluantes, mais la LMFNM peut entraîner une hausse de l’exposition, une baisse de la perception des méfaits, une hausse de la prévalence de la consommation de cannabis chez les mineurs par l’exemple d’autrui, par la normalisation du comportement au sein des ménages et dans la collectivité, ainsi qu’une hausse des taux de conduite avec facultés affaiblies par le cannabis, lesquels peuvent alourdir le fardeau des blessures de la route. L’expérience de la LMFNM dans les États américains montre une hausse des taux de visites à l’urgence pour des intoxications pédiatriques causées par l’ingestion involontaire d’aliments contenant du cannabis et pour des brûlures graves causées par des explosions durant l’extraction du cannabis à domicile. Les arguments à l’appui de la légalisation ont créé un cadre juridique strict pour resserrer le contrôle du cannabis au Canada, mais en raison des conséquences du cannabis pour la santé et la sécurité des enfants et des adolescents, il faudrait pousser la recherche, communiquer avec les responsables des politiques et les praticiens de la santé publique et offrir aux parents, aux proches aidants et aux jeunes une sensibilisation fondée sur les preuves.

## Introduction

Commencing October 2018, Canadians will witness a change in the national policy toward cannabis, which will see the legalization of non-medical marijuana (LNMM) with the passing of Bill C-45, The Cannabis Act (Task Force on Cannabis Legalization and Regulation 2016).

The potential impacts of legalization on the safety of children and youth merit serious debate, as the rate of cannabis use by Canadian adolescents is one of the highest among developed countries (UNICEF Office of Research 2013). Some experts believe that legalization in the context of a strictly regulated framework for controlling production, distribution, and sale can decrease harms, such as marginalization, stigmatization, and convictions for personal cannabis use. Current criminal penalties are considered to be disproportionately harsh and hamper future educational and career opportunities of young cannabis users without having an adequate deterrent influence. Moreover, legalization is expected to improve control over cannabis price, quality, potency, and access—especially by minors (Rehm and Fischer 2015).

Others argue, however, that valid comparative evidence does not exist to conclude that the current policy has been ineffective in promoting a safe community, as we are unable to predict the extent of cannabis use, nor the magnitude of consequences that would have occurred without current prohibitions. Moreover, it is argued that the state-controlled cannabis supply may contribute to the growth of a black market that targets young users of limited means to provide them with less expensive and low-quality products. In this latter perspective, major concerns regarding unfavourable public health impacts of legalization include lower perceived harm of use, higher rates of use, driving under the influence of cannabis (DUIC), road traffic injuries (RTIs), and healthcare utilization (Kalant 2015; Hajizadeh 2016).

In the midst of these uncertainties about the potential public health and safety impacts, the experiences of jurisdictions that are in the post-legalization period might serve to shed a light on the status quo. In 2012, Washington State (WA) and Colorado (CO) became the first states in the US to vote for LNMM. Approximately 9 months later, the proportion of drivers testing positive for THC (delta-9-tetrahydrocannabinol) in fatal crashes began to trend upward at an annual rate of 9.7% in WA. By 2014, this proportion was more than twice as high as the averages of the prior four years (Tefft et al. 2016). In CO, the number of cannabis-related fatal crashes also increased sharply and steadily (Colorado Department of Transportation nd), and the ED visits for marijuana exposure in patients aged 9 years and older nearly doubled following LNMM. The same increasing trend was observed in the number of calls to the Colorado Poison Control Center (Kim and Monte 2016). The available rates and trends, although alarming, are mixed and not conclusive (Compton 2017), and causality cannot be inferred. Still, the available evidence indicates grounds for concern as LNMM draws closer in Canada.

Amid the expressed concerns over the potential public health impacts of LNMM, child and adolescent safety issues are underrepresented. Currently, preventable injuries claim more lives than all other causes of death among children in Canada (Parachute 2015). This already significant burden justifies caution toward any policy that might lead to more pediatric injuries and deaths. Drawing on the experience of other jurisdictions that have legalized recreational cannabis use, the purpose of this commentary is to discuss the implications of legalization of recreational cannabis in Canada for unintentional injuries among children and adolescents. These potential implications and underlying mechanisms and pathways are discussed based on the review of published papers and reports.

## Implications of adult recreational cannabis use for pediatric injuries

The parent/caregiver role presents a mechanism by which LNMM can potentially harm children and adolescents. LNMM renders cannabis possession and use legal activities for adults (Rehm and Fischer 2015), who have a role in shaping the attitudes and behaviour of children and adolescents through supervision and role modeling (Committee on Substance Abuse and Committee on Adolescence 2015). Parental cannabis use is associated with adolescent lifetime and recent use (Vermeulen-Smit et al. 2015), as the behaviour is first taught and then normalized through repetition, potentially leading to reduced perception of harm. Thus, in the context of LNMM, recreational cannabis use among adults—and specifically parents—can bring about earlier initiation and higher prevalence of use by minors through a process of role modeling and normalization (McKee et al. 2018), thereby contributing to increased risk of preventable injuries associated with cannabis.

Having legal cannabis products at home also increases the risks associated with the products themselves. LNMM in CO was accompanied by an increased incidence of pediatric intoxication due to unintentional ingestion of edibles (cannabis-containing food products) and a significant increase in the mean rate of marijuana-related pediatric hospital visits (Monte et al. 2015). While Health Canada will regulate the packaging, labeling, and marketing of edible products to reduce their appeal and risk to children, an edible that is sold in a childproof container would be readily accessible to a child once the package is opened.

Delayed effect of edible consumption is another concern, which might prompt adolescents to increase the dose to feel the effect, compounding the risk of intoxication and poisoning. Further, variability in the effect of ingested cannabis makes it difficult to know how much of the product to use, even with standardized dosages and warnings on the package labels, and the fact that the effects linger much longer than smoking, contributing to the risk of an overdose or undertaking risky activities (such as driving) while intoxicated (Ghosh et al. 2015). Moreover, the proposed Cannabis Act allows for growing up to four cannabis plants per household (Task Force on Cannabis Legalization and Regulation 2016)—and making home-based cannabis food and drinks increases the risk of exposure to cannabis without any quality, safety, or potency monitoring.

While less has been said about the potential association between LNMM and increased risk of burns, there are serious concerns regarding electrical and fire hazards within houses associated with activities to process home-grown cannabis plants. Evidence from emergency rooms in CO shows a remarkable increase in the number of burns attributable to home-based extraction of butane hash oil following LNMM (Monte et al. 2015).

## Cannabis-impaired driving and road traffic injuries

RTIs are currently the leading cause of injury-related deaths among Canadian children (Parachute 2015). With legalization of cannabis—a substance with renowned psychoactive properties—there would be justifiable concerns about the potential impacts on this already substantial burden.

Robust evidence supports the increased risk of collisions following DUIC (driving under the influence of cannabis) (Asbridge et al. 2012). Cannabis compromises divided attention tasks, decision making, and responding to unexpected events, such as a pedestrian child darting out onto the street (Beirness and Porath-Waller 2017). Reports from jurisdictions now post-LNMM indicate concerns regarding the increasing trend of DUIC and cannabis-involved fatal crashes (Tefft et al. 2016; Colorado Department of Transportation nd). Due to the lack of sufficient evidence, there is uncertainty regarding the specific impact of LNMM on the rate of pediatric transport-related injuries as pedestrians, cyclists, and car occupants. Nevertheless, any policy that has potential to add to the currently high burden warrants additional emphasis on preventive action.

There are also raised concerns about young drivers who are in theory protected by the law of zero-tolerance for THC under the graduated driver licensing (GDL) program, but might generally be unaware of the risks associated with DUIC and be under the impression that the likelihood of getting caught and penalized for DUIC would be low (McKiernan and Fleming 2017). Policy and practice around DUIC within the context of LNMM need to address the specific risks to adolescent drivers and their peers, an already recognized vulnerable group, to avoid compromising the influence of the positive steps taken previously through GDL and roadside prohibitions for driving under the influence of alcohol.

As previously stated, due to limited reports from jurisdictions that have already experienced LNMM, there is no current evidence to support the impact on child and adolescent road safety; but this is undoubtedly an area that should be considered for ongoing monitoring, research, and preventive action.

## Conclusion

LNMM is a road less paved with scientific evidence regarding the public health benefits and harms of legalized cannabis use, and the American Academy of Pediatrics declared its opposition to LNMM within its policy statement while supporting decriminalization (Committee on Substance Abuse and Committee on Adolescence 2015).

Although the bulk of evidence from jurisdictions with similar policies is still limited, there is growing evidence to warrant concern that LNMM may contribute to the already high burden of unintentional injuries among Canadian children and adolescents. The main mechanisms and pathways of concern are increased availability; access and use through parental/familial role modeling; normalization of the behaviour; lower perceived harm; higher rates of DUIC and by extension, higher RTIs among pediatric car occupants and vulnerable road users (pedestrians and cyclists); poisoning and intoxication; and burns.

While the justification for legalization has created a strict legal framework for improved control of cannabis in Canada, the implications for health and safety of children and adolescents necessitate further study, communication with policy-makers and public health practitioners, together with evidence-based education of parents, caregivers, and youth.
